# Selection and Drift: A Comparison between Historic and Recent Dutch Friesian Cattle and Recent Holstein Friesian Using WGS Data

**DOI:** 10.3390/ani12030329

**Published:** 2022-01-29

**Authors:** Ina Hulsegge, Kor Oldenbroek, Aniek Bouwman, Roel Veerkamp, Jack Windig

**Affiliations:** 1 Animal Breeding and Genomics, Wageningen University & Research, P.O. Box 338, 6700 AH Wageningen, The Netherlands; kor.oldenbroek@wur.nl (K.O.); aniek.bouwman@wur.nl (A.B.); roel.veerkamp@wur.nl (R.V.); jack.windig@wur.nl (J.W.); 2 Centre for Genetic Resources, The Netherlands, Wageningen University & Research, P.O. Box 338, 6700 AH Wageningen, The Netherlands

**Keywords:** genetic diversity, Dutch Friesian, Holstein Friesian, cattle breeds, WGS

## Abstract

**Simple Summary:**

Over the last century, genetic diversity in the cattle species has been affected by the replacement of many local, dual-purpose breeds with a few specialized, high-output dairy breeds. This replacement caused a sharp decline in the population size of local breeds. In the Netherlands, the local Dutch Friesian breed has gradually been replaced by the Holstein Friesian. This resulted in a rapid decrease in numbers of the Dutch Friesian breed with an associated risk of loss of genetic diversity due to drift. The objective of this study is to investigate genomewide genetic diversity between a group of historic and recent Dutch Friesian bulls and a group of recently used Holstein Friesian bulls. Our findings showed that a large amount of diversity is shared between the three groups, but each of them has some unique genetic identity (12% of the single nucleotide polymorphism were group-specific). The genetic diversity of the Dutch Friesians reduced over time, but this did not lead to higher inbreeding levels—especially, inbreeding due to recent ancestors has not increased. Genetically, the recent Dutch Friesians were slightly more different from Holstein Friesians than the historic Dutch Friesians. Our results also highlighted the presence of several genomic regions that differentiated between the groups.

**Abstract:**

Over the last century, genetic diversity in many cattle breeds has been affected by the replacement of traditional local breeds with just a few milk-producing breeds. In the Netherlands, the local Dutch Friesian breed (DF) has gradually been replaced by the Holstein Friesian breed (HF). The objective of this study is to investigate genomewide genetic diversity between a group of historically and recently used DF bulls and a group of recently used HF bulls. Genetic material of 12 historic (hDF), 12 recent DF bulls (rDF), and 12 recent HF bulls (rHF) in the Netherlands was sequenced. Based on the genomic information, different parameters—e.g., allele frequencies, inbreeding coefficient, and runs of homozygosity (ROH)—were calculated. Our findings showed that a large amount of diversity is shared between the three groups, but each of them has a unique genetic identity (12% of the single nucleotide polymorphisms were group-specific). The rDF is slightly more diverged from rHF than hDF. The inbreeding coefficient based on runs of homozygosity (Froh) was higher for rDF (0.24) than for hDF (0.17) or rHF (0.13). Our results also displayed the presence of several genomic regions that differentiated between the groups. In addition, thirteen, forty-five, and six ROH islands were identified in hDF, rDF, and rHF, respectively. The genetic diversity of the DF breed reduced over time, but this did not lead to higher inbreeding levels—especially, inbreeding due to recent ancestors was not increased.

## 1. Introduction

Over the last century, genetic diversity in many European national cattle populations has been affected by replacement of traditional local breeds with just a few specialized milk producing breeds, e.g., the Holstein Friesian, Brown Swiss, and Jersey. The predominant use of these breeds caused a sharp decline in the population size of local dual-purpose breeds [1,2]. Although less productive under intense production conditions, these local breeds may carry alleles that enabled them to adapt to local conditions. Moscarelli et al. [3] reported the presence of several genomic regions that vary between original and modern Brown cattle populations, in line with their different breeding histories. Selection and genetic drift will both have contributed to the genetic differentiation between original and modern breeds. Therefore, local breeds might represent an important genetic resource to facilitate animal breeding when changes occur in production systems and market requirements.

The change in use from local breeds to specialized breeds has been observed in many industrialized European countries including the Netherlands [4,5]. Until 1975, the Dutch Friesian cattle (DF) dominated the Dutch national cattle population (76%) [6,7]. By the late nineteenth century, the Dutch Friesians were internationally known as exceptionally productive dairy cattle. American dairy farmers imported them in the 1870s and 1880s for this reason [8]. In the United States, where their progeny became known as Holstein Friesians, the farmers continued to breed them as high-yielding dairy cows [8]. In the Netherlands, there was emphasis on conformation and beef production in addition to milk production, because the Dutch Friesians were kept as a dual-purpose breed. Since the 1960s and 1970s, Holstein Friesians (bulls, semen, and embryos)—descendants of the original Dutch Friesian cattle—were imported from the United States into the Netherlands and used to improve the genetic ability for milk production. Consequently, DF in the Netherlands has gradually been replaced by Holstein Friesian (HF) during the past decades. Currently, more than 90% of the Dutch dairy cattle population consists of HF [6,9]. This upgrading process resulted in a rapid decrease in numbers and, therefore, a potential loss in genetic diversity due to drift in the DF breed [10]. Since the beginning of the 1960s, genetic material from Dutch local cattle breeds and later from the HF as well has been collected and stored in the Dutch gene bank. Stored material contains genetic diversity of the breed at the time of sampling, which may include diversity that, since then, has been lost in situ due to selection and genetic drift.

Currently, single nucleotide polymorphism (SNP) chips are available for the majority of livestock species, targeting genetic variants widely spread along their entire genomes. Importantly, SNPs detected in commercial breeds were selected and used to design the chips leading to some ascertainment bias when using these chips in studies with local breeds [11]. The latest advances and increasing economic accessibility of whole-genome sequencing (WGS) brings new perspectives exploring the genetic information of local breeds. Unlike SNP chips, WGS is the complete genome sequence containing all polymorphisms present on the genome. Thus, WGS does not have the problem of ascertainment biases. Another advantage of WGS is that it contains information on rare variants [12] and, additionally, maps genomic regions highly affected by selection pressure [13]. WGS enables the estimation of relationships between individuals more accurately because it is based on both common and rare variants. Furthermore, WGS has information on common variants in local breeds, which might be rare or absent in specialized dairy breeds, such as Holstein Friesian. However, to date, much of the effort has been devoted to dominant commercial breeds, with local breeds rarely studied. Furthermore, changes to genetic diversity in breeds over time is also rarely studied.

The objective of this study is to investigate genomewide genetic diversity and loss of alleles between three groups of bulls, chosen from the historic (1961–1989) and recent (2003–2015) DF population and the recent HF (1998–2014) population. Differences in allele frequencies and in homozygosity will provide insights into the mechanisms underlying their genomic differences caused by selection or a sharp decrease in the number of breeding animals.

## 2. Materials and Methods

### 2.1. Animals

Genetic material from purebred Dutch Friesian animals born from 1961 onwards has been preserved by the Centre for Genetic Resources, the Netherlands (CGN) of Wageningen University and Research, i.e., the Dutch Gene bank (https://www.genebankdata.cgn.wur.nl/, accessed on 27 July 2021. From this genetic material, a group of 12 historic (1961–1989; hDF) and a group of 12 recent (2003–2015; rDF) Dutch Friesian bulls were sequenced. The animals were selected based on their year of birth by taking the oldest and youngest DF sires, while avoiding closely related animals in the selection. Furthermore, sequence data from a group of 12 recently used Holstein Friesian bulls in the Netherlands (1998–2014; rHF) were available for this study. These bulls were a selection of unrelated animals born in different years and sequenced for a project in 2017 on efficiency and health indices of the breeding company in dairy cattle CRV.

### 2.2. Short Read Sequencing Mapping and Variant Calling

DNA was isolated form sperm using the Echolution Sperm DNA kit (BioEcho Life Science GmBH, Köln, Germany). Library preparation and sequencing of the DF animals were performed at the Institute national de la Recherche Agronomique (INRA), France, following their established protocols. Library preparation and sequencing of the rHF animals were performed at BGI, China, following their established protocols. Paired-end sequencing was performed on the Illumina HiSeq platform. All animals were sequenced with short reads at 10× coverage. We followed the 1000 Bull Genomes Project Run 7 guideline (1000 bulls GATK fastq to GVCF guidelines; version: 18 June 2018) to process the raw sequence data into both binary alignment map (BAM) and genomic variant call format (GVCF) files [14]. A per-base sequence quality, for the raw sequence reads, was examined using the fastQC software (version: 0.11.7) [15]. The reads were trimmed and filtered using Trimommatic (version: 0.38) [16] and then mapped against the bovine reference genome ARS-UCD1.2_Btau5.0.1Y (version: 8 May 2018) using the Burrows–Wheeler Aligner (BWA; version: 0.7.17) [17]. Samtools (version: 1.8) [18] was used to sort the BAM files and create index files. Polymerase chain reaction (PCR) duplicates were identified using the ‘MarkDuplicates’ function of Picard (version: 2.18.2) software (http://broadinstitute.github.io/picard, accessed on 27 July 2021). Base quality recalibration (BQSR) was performed with ‘BaseRecalibrator’ and ‘PrintReads’ of the Genome Analysis Toolkit (GATK; version: 3.8-1-0-gf15c1c3ef). The known variants file (ARS1.2PlusY_BQSR.vcf.gz; version: 15 June 2018) generated by the 1000 Bull Genomes Project was used to mask out positions with known variation to avoid confusing real variation with errors. The before/after BQSR reports were checked using ‘AnalyzeCovariates’ to ensure that base quality scores were corrected as expected. SNPs were called using the GATK ‘HaplotypeCaller’ with ‘-ERC GVCF’ option. The rate of genome alignment and average sequencing depth were determined with Qualimap (version 2.2.1) software [19]. The ‘GenotypeGVCFs’ arguments of GATK were used to identify variants simultaneously in all samples. ‘VariantRecalibration’ and ‘ApplyRecalibration’ were used to produce filtering information for SNPs. The process has been shown to outperform the ‘hard’ filtering of variants [20]. For the recalibration steps, the truth and training datasets described by Jagt et al. [21] were used, replacing the Run6 datasets of the 1000 bulls by datasets of Run 7.

The variants were called across all three groups combined. Only biallelic SNPs were kept, and filtration for Minor Allele Frequency was not applied at this stage. Per group (hDF, rDF, and rHF), a maximum of 2 out of 12 animals were allowed to have a missing value per SNP. These criteria resulted in a total of 10,780,681 SNPs, genotyped in all three groups, for further analysis.

### 2.3. Group Structure and Identification of Group-Specific SNPs

To identify group-specific SNPs, each SNP that passed the applied filtering criteria was analyzed according to the information about the three groups. An allele was labelled as group-specific if it was only present in one of the three groups and not detected in any of the other two, also called private allele [22].

To explore the genetic distance between animals of the three groups, a Principal Component Analysis (PCA) was performed using the --pca function in PLINK (version: 1.90) [23]. The graphical representation was depicted using the statistical R software (http://www.R-project.org/, accessed on 4 November 2021).

### 2.4. Genetic Diversity Parameters

Various parameters were used to estimate genetic diversity within the groups: Observed (H_o_) and Expected Heterozygosity (H_e_) and Minor Allele Frequency (MAF). Observed and Expected Heterozygosity were calculated using VCFtools (version 0.1.13) (--het) [24]. The MAFs were calculated using the—freq option in PLINK.

### 2.5. Selection Signature Analysis

The fixation index (Fst) was used to characterize the differentiation between the groups, i.e., to identify selection signatures. The pairwise estimates of Fst among the three groups (hDF-rDF, hDF-rHF, and rDF-rHF) were calculated using VCFtools with the Weir and Cockerham approach (--weir-fst-pop) [25]. Windows of SNPs were used to minimize the stochastic effect of a single SNP. The Fst values were averaged across 40-kb windows, with a sliding frame of 20 kb at a time. The parameters for the VCFtools program were “—fst-window-size 40,000--fst-window-step 20,000”, following Rafiepour et al. [26]. To normalize the mean *Fst* values, Z-transformation was performed $ZFst=\frac{Fst-\mu Fst}{aFst}$, where *Fst* is mean *Fst* in a window, *µFst* is an average *Fst* over all windows, and *αFst* is a standard deviation of *Fst* values of all windows tested for a given comparison [27]. The ZFst was visualized in the form of a Manhattan plot by the R package ‘qqman’ (version 0.1.8) [28]. Candidate genomic regions under selection were defined as regions where the *ZFst* value > 8. To reduce the number of false positives, windows with less than 5 SNPs were removed.

### 2.6. Measure of Runs of Homozygosity

Runs of homozygosity (ROHs) were calculated to identify contiguous regions of the genome where an animal is homozygous across sites. ROHs were calculated individually using PLINK with adjusted parameters: --homozyg --homozyg-window-snp 50 --homozyg-snp 50 --homozyg-kb 300 --homozyg-density 50 --homozyg-gap 1000 --homozyg-window-missing 5 --homozyg-window-threshold 0.05 --homozyg-window-het 3, following Cheng et al. [29]. No linkage disequilibrium (LD)-based pruning was performed before calculating ROHs. Individual degree of inbreeding based on ROH analysis (Froh), genomewide as well as chromosome-wide, was calculated using the function Froh_inbreeding of the R package ‘detectRUNS’ (version 0.9.6) [30]. In addition, Froh (Froh > 2 Mb, Froh > 4 Mb, Froh > 8 Mb, and Froh > 16 Mb) derived from ROHs of different length (>2, >4, >8, and >16) were calculated.

### 2.7. Runs of Homozygosity Islands

To identify genomic regions most commonly associated with ROH, i.e., ROH Islands, the percentage of the occurrences of a SNP in ROH was calculated by counting the number of times the SNP was detected in those ROHs across animals [31] within each group using the R package ‘detectRUNS’. The most common runs were retrieved using the function ‘tableRuns’ of the package ‘detectRUNS’ with a threshold value of 0.8. This means that a ROH has to be present in at least 80% (10 of the 12 animals) of each group hDF, rDF, and rHF to be included in a ROH island.

## 3. Results

### 3.1. SNP Distribution

**Figure 1:**
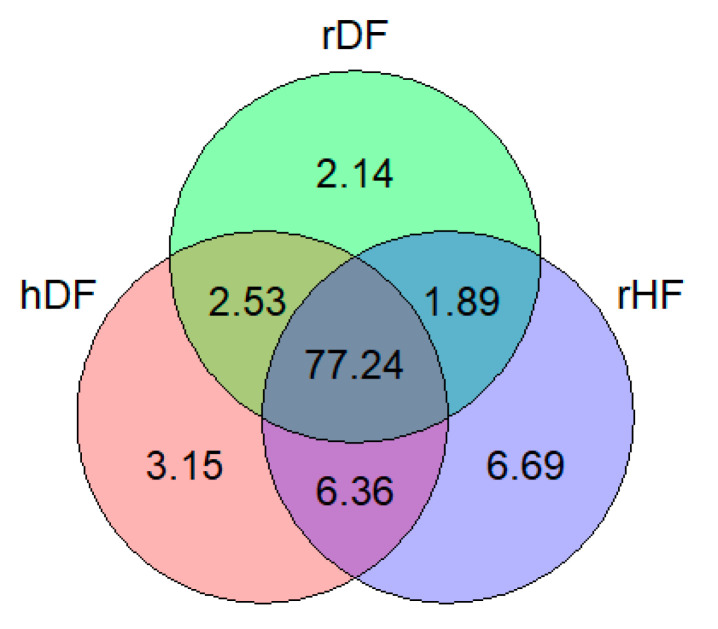
Venn diagram showing percentage of shared and group-specific variants in each group.

Among the set of 10,780,681 SNPs, 11.98% were identified as putatively group-specific (Figure 1), indicating that the genotype of one of the alleles was present in only one of the three groups (hDF, rDF, and rHF). SNPs specific for rHF were the most abundant (6.69%), while rDF displayed the lowest number of group-specific SNPs (2.14%). It is notable that the percentage of group-specific SNPs for the DF (hDF and rDF) groups was 7.82%, which is slightly higher as for rHF (6.69%). Over 77% of the SNPs occurred in all three groups.

**Figure 2:**
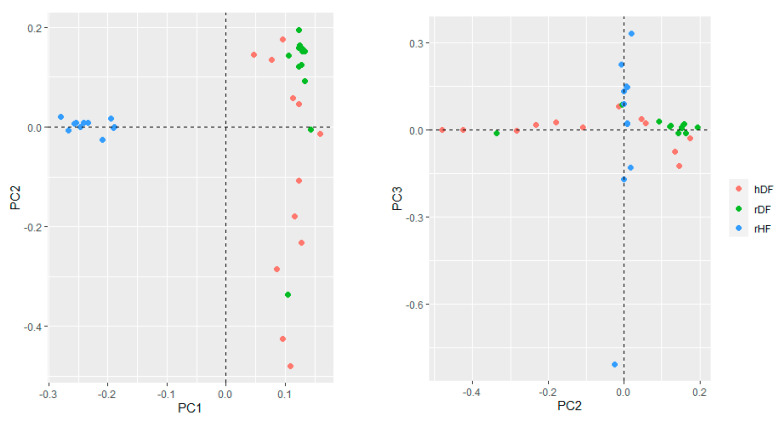
Genetic relationships based on PCA between the three groups.

The genetic structure of the three groups assessed with the first three principal components of PCA accounted for 13.5% (PC1), 6.4% (PC2), and 5.6% (PC3) of the total variation (Figure 2). The first principal component (PC1) distinguished hDF and rDF from rHF. The hDF group differentiated across the second principal component (PC2), while rDF grouped more together, although overlapping with hDF. One rDF animal was positioned away from the other rDF animals along PC2. The third PC distinguished variation within the rHF, indicating one sire more distantly from the others.

### 3.2. Genetic Diversity Parameters

Minor Allele Frequency together with Observed and Expected Heterozygosity (H_o_ and H_e_), were used to determine the levels of genetic variability in the three groups (Table 1). The average MAF and H_e_ were almost similar for the three groups (MAF: 0.16; H_e_: 0.25). For all three groups, H_o_ was lower than H_e_. There were small differences in H_o_ between three groups: rHF had the lowest H_o_ value (0.19) and rDF had the highest H_o_ value (0.20).

Genetic differentiation among the three groups ranged from low to moderate, as indicated by the weighted pairwise Fst values that ranged from 0.01 to 0.11 (Table 2). Recent and historic DF were genetically very similar, and somewhat different from rHF.

### 3.3. Genomic Inbreeding Coefficients

The inbreeding coefficient derived from ROH (Froh) in different length categories differentiated past and recent inbreeding (Table 3). Recent DF tended to have a larger fraction of the genome covered by ROH compared with hDF and rHF. The general average inbreeding coefficient was significantly higher for rDF (0.24) than rHF (0.13) (*p* < 0.05). The level of ancient inbreeding reached 0.05–0.13 (Froh > 2 Mb) with rDF having the highest level, whereas the recent inbreeding load was 0.01 (Froh > 16 Mb) for all three groups. So, Froh decreased as the minimum length of the ROH increased.

The mean Froh values for each chromosome followed the same pattern as those computed for the whole genome, but there was variation across the chromosomes (Additional file 3, Figure S1). For most chromosomes, the mean Froh was highest for rDF and lowest for rHF. Only chromosome 25 showed lower values for rDF compared with hDF and rHF. The chromosomal Froh variability within groups was high.

### 3.4. Measure of Runs of Homozygosity

**Figure 3:**
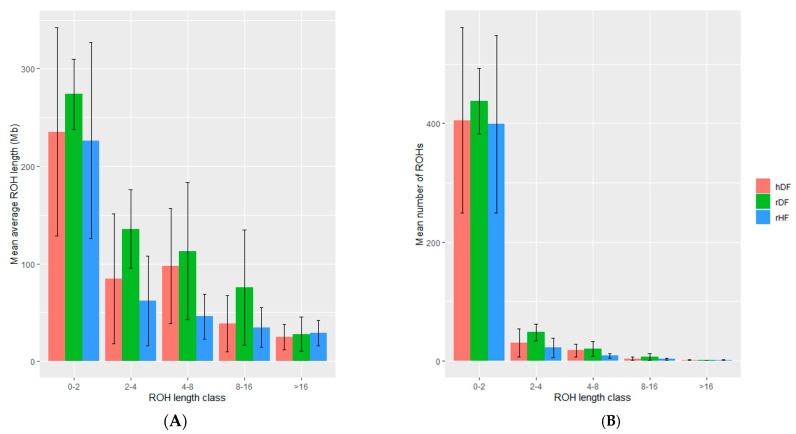
The mean and standard deviation of the average length of runs of homozygosity (ROH) (**A**) and mean number of ROH within each ROH length class (**B**).

The number and length of ROHs differed among animals and across groups (Table 4). The rDF group had the highest number of ROHs (513), whilst rHF had the lowest number (424). Additionally, rDF had the highest average length of ROHs (1.19 Mb) and rHF the lowest (0.73 Mb). Variation existed in the distribution of the various ROH length classes, but a common pattern was observed across the groups (Figure 3, Figure S1). The majority of ROH segments (~85 to 95%) is found in the length class 0 to 2 Mb for all three groups. The number of ROHs was the highest for rDF and the lowest for rHF in all classes, except class >16 Mb. The range of number of ROHs was more variable in the hDF and rHF groups in comparison with a more even number of ROHs for rDF group.

### 3.5. Genomewide Selection Signature Analysis

**Figure 4:**
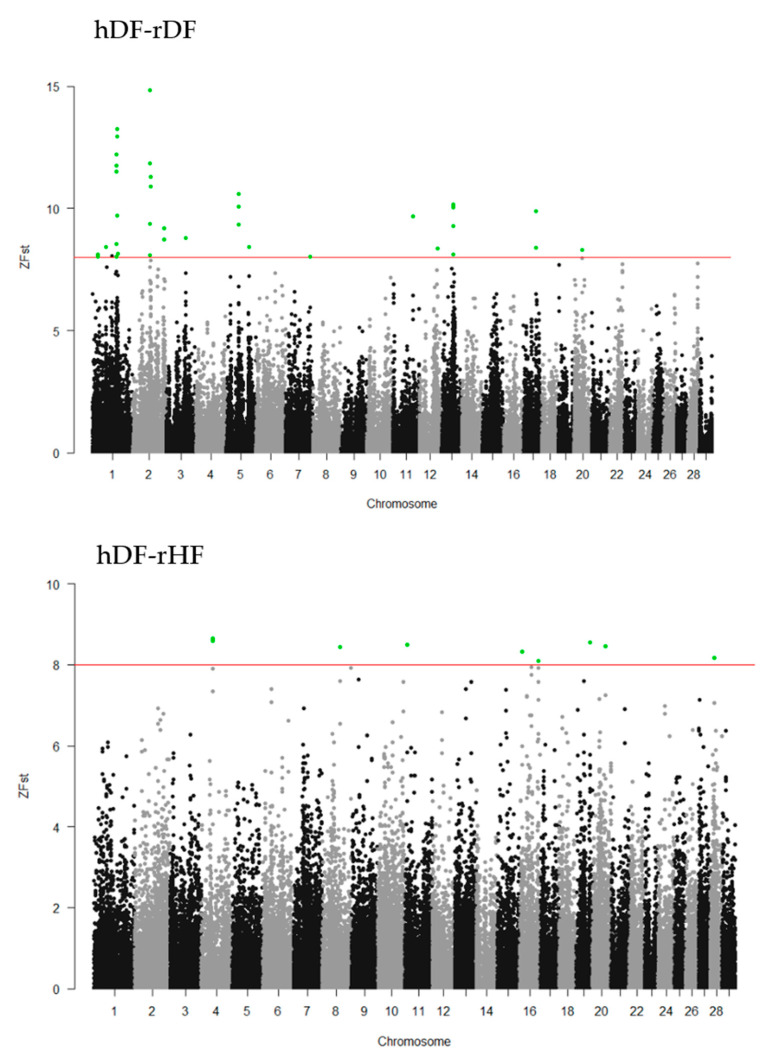

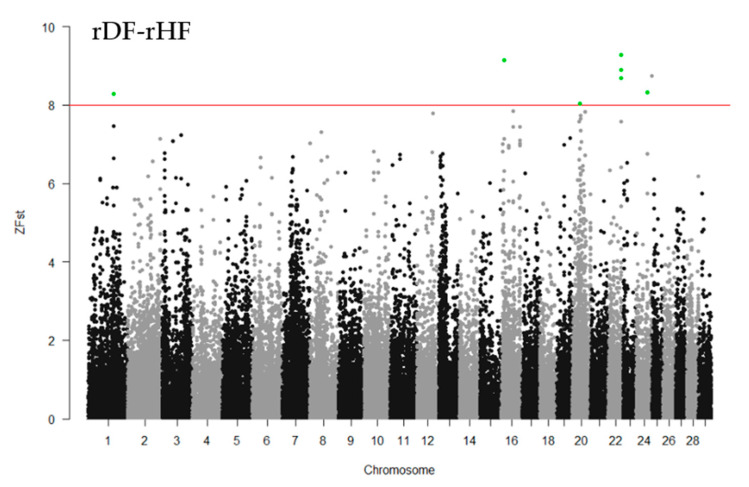
Manhattan plots of Z-transformed fixation index (ZFst) across all autosomes. The ZFst values were calculated for each sliding 40-kb window with steps of 20 kb across all autosomes. The solid red line indicates ZFst values > 8; differentiated genomic regions (ZFst values > 8 and the number of SNPs in the region > 5) are highlight green.

To identify the differentiated genomic regions among the groups, the Z-transformed Fst (ZFst) values based on SNPs in 40-kb sliding windows with 20-kb steps were calculated. The ZFst varied markedly across the genome in all three comparisons (hDF-rDF, hDF-rHF, and rDF-rHF) (Figure 4). We identified highly differentiated genomic regions (ZFst > 8) across autosomal chromosomes, i.e., thirty-eight for hDF versus rDF, nine for hDF versus rHF, and seven for rDF versus rHF (Figure 4, Additional file 1: Table S1). For the comparisons between hDF and rDF, the strongest differentiated genomic regions were detected on BTA1 (96.50–96.58 Mb and 98.80–98.86 Mb) and BTA2 (72.54–72.62 and 75.80–75.86 Mb). For hDF–rHF comparison, the strongest differentiated region was detected on BTA4 (44.66–44.72 Mb). In the case of the rDF versus rHF group, the strongest differentiated regions were located on BTA1 (101.26–101.30 Mb), BTA16 (9.88–9.92 Mb), BTA20 (28.84–28.88 MB), BTA22 (three regions between 52.80–52.88 Mb), and BTA24 (44.16–44.20 Mb).

### 3.6. Runs of Homozygosity Islands

The genomic distribution of ROH islands was nonuniform across chromosomes, regardless of the group (Additional file 2: Table S2). Differences in the segments of ROH islands on the chromosomes were identified between the three groups. In total, we identified thirteen, forty-five, and six ROH islands for hDF, rDF, and rHF, respectively. Almost all ROH islands found in hDF overlapped with ROH islands found in rDF. No genomic regions were common to all the three groups.

Only one of the ROH islands identified ((BTA1: 101.26–101.30 Mb in rDF) overlapped with the genomic regions identified using pairwise Fst (hDF vs. rDF and rDF vs. rHF).

## 4. Discussion

### 4.1. General

In this study, we investigated genomewide genetic diversity within and between groups of historic and recent DF bulls, and a group of recent HF bulls. In the Netherlands, local, dual-purpose cattle breeds, including the DF breed, have gradually been replaced by the specialized dairy breed HF during the past decades. This has caused a decline in the population size of local breeds and potentially a loss of genetic diversity. The historic Dutch Friesian animals used in this study were born between 1961–1989, when the population size of DF was still large. The recent DF animals were born between 2003 and 2015. In the approximately 5–10 generations between hDF and rDF, the population size (number of adult cows) of DF has declined significantly, from 629,410 in 1970 to 3153 in 2017 [6]. DF is now classified as being at risk (https://www.fao.org/dad-is; accessed on 15 October 2021).

### 4.2. Divergence between Groups

Our findings indicate that a large amount of diversity is common to the three groups. A high percentage of shared SNPs was found for the 3 groups, which is expected since all groups descend from the same ancestors. The founders of the Holstein Friesian breed originated from the Dutch Friesian breed [32]. Furthermore, all three groups have a small number of group-specific SNPs (2–7%), indicating that each has some unique genetic identity. The PCA analysis displayed that the rDF group has diverged slightly from hDF group over the last approximately 5–10 generations, presumably as a result of genetic drift. Genetically, DF is distinct from HF, probably resulting from the selection of HF as a specialized dairy breed, whereas farmers aimed to maintain DF as a dual-purpose breed. The genetic distinction between DF and HF is in agreement with results reported by van Breukelen et al. [6] and Hulsegge et al. [33]. Likewise, a PCA analysis separated the Swedish Holstein Friesian breed from native Swedish cattle breeds [34]. In our study, the results of the PCA are confirmed by the pairwise Fst. Although the DF and HF groups are selected for different purposes, we expected some similarities between them, and these are indicated in this study by the moderate average Fst values (0.1). A similar pairwise Fst value between DF and HF, based on SNP array data and a larger number of animals, was reported by Hulsegge et al. [35].

### 4.3. Genetic Diversity within Groups

A decimation in numbers of a population is expected to reduce its genetic diversity and increase inbreeding levels. Indeed, diversity in the DF has reduced, e.g., rDF contains fewer specific alleles than hDF and, in the Principal Component Analysis, members of hDF are spread out across PCA2 while rDF animals cluster. However, based on manually checking the individual pedigrees of the hDF and rDF group, inbreeding levels have not increased. On the contrary, H_o_, an indication of a lack inbreeding has increased in rDF. This is confirmed by the inbreeding level determined by pedigree for the whole population [36], which increased initially from around 3% in 1990 to above 5% in 2005 and decreased since then to under 4% in 2020; in 1970, the average inbreeding level was around 0% [37]. One explanation for lower Observed Heterozygosity than expected is local inbreeding.

Manual checking of individual pedigrees indicated local breeding in historic DF. Breeders generally used their own bulls and certainly no bulls from other regions. In particular, there was a separation between Friesian bulls and bulls from the North Holland region. The animals from North Holland were, for example, slightly larger and produced more milk, but had less conformation than the animals from Friesland [38]. Currently, this separation has largely disappeared, and most animals have similar ancestry. However, one breeder went against the tide and eliminated from his stock all influence of an ancient Friesian bull who is ancestor to most other animals in the breed (pers. comm. Henk Sulkers). The deviating bull in rDF in PCA2 was bred by this breeder.

In the 1990s, when the DF rapidly declined, the DF herdbook initiated a strategy called fundament breeding to counter the loss of diversity. In this strategy, the breed is divided into several fundaments, each consisting of one or a few herds. Within each fundament, 4–5 own bulls are used and rotated over groups of cows so that inbreeding is postponed for at least three generations. Bulls should not be exchanged across fundaments to safeguard their genetic distinctness. This latter point was not strictly adhered to [37] and our data show no clear separation of fundaments in rDF; however, the strategy to postpone inbreeding seems to have worked. Although ROH levels are higher in rDF, this is due to ROH segments of shorter length only. These shorter segments indicate inbreeding due to ancestors further back in the pedigree.

In conclusion, although the diversity has reduced, this has not led to higher inbreeding levels—particularly, inbreeding due to recent ancestors has not increased. The policy of the breeding organization has influenced inbreeding levels but has not prevented the loss of some diversity, and diversity conserved in the gene bank has been lost from the live population. Therefore, to maintain and improve the genetic diversity in the current DF population, material from historic individuals present in the gene bank, should be used in the life population. Furthermore, the current strategy of rotating bulls within the fundaments should be maintained to limit the increase in inbreeding.

### 4.4. Differentiated Genomic Regions

The pairwise Fst highlighted the presence of several genomic regions that differentiated between the groups. The Fst-based approach does not directly indicate in which group selection is operating. In this study, the region with the highest ZFst values for the comparison hDF–rDF are observed from BTA1 (96.50–96.58 and 98.80–98.86 Mb) and BTA2 (72.54–72.62 and 75.80–75.86 Mb). In two of the four regions, no genes are located, while three genes are located in the other two regions: EIF5A2, RPL22L1, and ENSBTAG00000051422. EIF5A2 is associated with fertility traits. EIF5A2 has been reported as a candidate gene for age at sexual maturity in Indian Buffalo [39] and for infertility in human [40]. RPL22L1 is also described as a candidate gene for age at sexual maturity in Indian Buffalo [39]. Furthermore, RPL22L1 is reported as associated gene in low-fertility buffalo bull spermatozoa [41]. This gene is also mentioned as a candidate gene for birth weight in Holstein Friesian [42,43]. This is in agreement with Estimated Breeding Values (EBV) for DF reported between 1980 and 2020, which indicate a decrease in fertility and birth weight [44]. For the hDF–rHF comparison, we detected the strongest signal on BTA4 (44.66–44.72 Mb). In this region, the gene RELN is located. As stated by Cerri et al. [45], RELN is involved in the regulation of pregnancy and lactation in Holstein cows. The latter is also reported by Lonergan et al. [46]. Furthermore, RELN affected aggressive behavior in pigs [47].

In the case of the rDF versus rHF group, we identified seven highly differentiated regions. Genes are only found in two regions on BTA22: ALS2CL, LRRC2, and TDGF1.

### 4.5. Runs of Homozygosity Detection and Distributions

Almost all ROH islands found in hDF partially overlapped with ROH islands found in rDF. These partially overlapped regions probably preserve segments in high homozygosis, characteristic of the ancient selection of the population. In these regions, several known candidate genes, such as HCHD7, FBXO2, MAD2L2, MOS, and PLAG1, are mapped (Additional file 2: Table S2). These candidate genes are predominantly related to biological regulation (32.8% of the candidate genes) and metabolic processes (26.2% of the candidate genes (http://www.pantherdb.org/ accessed on 24 October 2021). Some traits are associated with these candidate genes as well. For example: the PLAG1-CHCHD7 region (BTA14: 23.33–3.38 Mb) is associated with stature; body size, including height; and weight in many cattle breeds [48,49,50,51,52].

The largest ROH island in the rDF group was found on BTA7 between 50.03–50.86 Mb. This region seems to coincide with an ROH island reported for taurine and indicine cattle breeds by Sölkner et al. [53] and for eight Chinese local cattle breeds reported by Xu et al. [54]. There are 15 candidate genes located within this ROH island: CTNNA1, DNAJC18, ECSCR, LRRTM2, MATR3, MZB1, PAIP2, PROB1, SIL1, SLC23A1, SMIM33, SNORA74, SPATA24, STING1, UBE2D2. Among them, we highlight the CTNNA1 gene, which has been associated with muscle development, skeletal muscle growth, and meat tenderness [55,56].

The largest ROH island in the rHF group was found on BTA8 between 105.89–106.21 Mb. This region contains one gene: ASTN2. The ASTN2 gene has been related to carcass weight of cattle [57] and meat traits in pigs [58].

### 4.6. Gene Bank

Our results revealed that the Dutch national genebank has stored material containing genetic diversity that has been lost in vivo by selection and drift. Gene bank collections have been shown to capture more diversity than some in situ populations thanks to periodic resampling [59,60,61]. It is also important that the gene bank pool stores genetic variation existing in the whole population. Van Breukelen et al. [6] reported that within the DF populations there are fundamental breeding groups, which have a unique genetic diversity. For pigs, Hulsegge et al. [62] reported that merging of commercial Landrace lines has reduced the genetic diversity of the Landrace population in the Netherlands, although a large proportion of the original variation is maintained. This stresses the value of gene banks to record and preserve variation that is lost in the process of merging lines, even over short periods of time.

### 4.7. Limitation of the Study

The accuracy with which allele frequencies and, therefore, inbreeding is estimated will depend on the sample size and number of SNPs [63]. In this study, we used 12 animals per group, which may have influenced the results. Although the sample size is small, it has previously been shown that a small sample size does accurately estimate population parameters when a large number of SNPs are used [64]. Our study contains a large number of SNPs (*n* = 10,780,681). According to Willing et al. [65] and Nazareno et al. [64], Fst can be accurately calculated based on small sample sizes (as small as *n* = 4 to 6) if the number of markers examined is large, i.e., larger than 1000. A small sample size can lead to poor population structure estimates, which affects the ability to differentiate between loci that were under selection and neutral population structure [66]. However, in our study, 12 animals per group were used, in line with a previous study that suggested that detecting regions under selection with Fst methods requires at least 10 samples [65].

## 5. Conclusions

Through the present study with WSG data, we have described the genetic differences between historic and recent Dutch Friesian groups, and a recent Holstein Friesian group. Our findings revealed that a large amount of diversity is shared in the three groups and each of the groups has a small number of group-specific SNPs. The two DF groups are genetically distinct from the HF group. rDF is slightly more diverged from HF than hDF. We identified changes in the genetic composition of the DF population in the approximately 5–10 generations between the historic and the recent DF group. The genetic diversity has reduced and a more homogeneous group has emerged. Although diversity was reduced, this did not lead to higher inbreeding levels—especially, inbreeding due to recent ancestors has not increased.

## Figures and Tables

**Table 1:** Genetic diversity parameters of within-group diversity of hDF, rDF, and rHF (mean ± standard deviation).

Group	Abbreviation	MAF	H_o_	H_e_
Historic Dutch Friesian	hDF	0.165 ± 0.152 ^a^	0.195 ± 0.025	0.250 ± 0.0005 ^a^
Recent Dutch Friesian	rDF	0.164 ± 0.155 ^b^	0.201 ± 0.015	0.250 ± 0.0003 ^a^
Recent Holstein Friesian	rHF	0.161 ± 0.153 ^c^	0.188 ± 0.035	0.249 ± 0.0016 ^b^

^a, b, c^ Different letters within a column indicates significant differences at *p* < 0.05.

**Table 2:** Estimated pairwise Fst (fixation index) as a measure of genetic differentiation between the three groups (Weir and Cockerham mean Fst, above diagonal; Weir and Cockerham weighted Fst, below diagonal; hDF, historic Dutch Friesian; rDF, recent Dutch Friesian; rHF, recent Holstein Friesian).

	hDF	rDF	rHF
hDF	-	0.0005	0.0624
rDF	0.0100	-	0.0719
rHF	0.0978	0.1105	-

**Table 3:** Mean and standard deviation of inbreeding coefficients (Froh) calculated from runs of homozygosity (ROH) with minimum length of 2 (ROH > 2), 4 (ROH > 4), 8 (ROH > 8), and 16 (ROH > 16) Mb for the three groups. Between brackets is the number of animals in the classes.

	Froh				
Group	General Mean	ROH > 2 Mb	ROH > 4 Mb	ROH > 8 Mb	ROH > 16 Mb
Historic Dutch Friesian	0.169 ± 0.095 ^ab^ (12)	0.081 ± 0.061 ^ab^ (11)	0.058 ± 0.034 (9)	0.019 ± 0.012 (9)	0.010 ± 0.005 (3)
Recent Dutch Friesian	0.243 ± 0.062 ^a^ (12)	0.132 ± 0.066 ^a^ (12)	0.078 ± 0.055 (12)	0.035 ± 0.028 (11)	0.011 ± 0.007 (5)
Recent Dutch Friesian	0.130 ± 0.067 ^b^ (12)	0.047 ± 0.036 ^b^ (10)	0.031 ± 0.022 (7)	0.019 ± 0.013 (5)	0.012 ± 0.005 (2)

^a, b^ Different letters within a column indicates significant differences at *p* < 0.05.

**Table 4:** Summary of specific regions of homozygosity (ROHs) in the three groups.

Group	# Animals	Number of ROH		Total ROH Length (Mb)	Average ROH Length (Mb)
		Mean ± sd	Range	Mean ± sd	Mean ± sd
hDF	12	449.75 ± 180.96	132–745	421.04 ± 235.43 ^ab^	0.87 ± 0.31 ^a^
rDF	12	513.50 ± 44.14	421–570	603.54 ± 154.92 ^a^	1.19 ± 0.34 ^b^
rHF	12	424.00 ± 161.48	75–653	323.92 ± 166.72 ^b^	0.73 ± 0.22 ^a^

^a, b^ Different letters within a column indicates significant differences at *p* < 0.05.

## Data Availability

The data presented in this study from The Centre for Genetic Resources, the Netherlands (CGN) of Wageningen University and Research will be online available before December 2022. Availability of the data of the “Melkveefonds” (HF sequences) are restricted to be used only for the current study, and thus, are not publicly available.

## Supplementary Materials

The following supporting information can be downloaded at: https://www.mdpi.com/article/10.3390/ani12030329/s1, Table S1: Differentiated genomic regions (ZFst > 8) across autosomal chromosomes for the hDF versus rDF population, the hDF versus rHF population and the rDF versus rHF population; Table S2: Genomic regions with the highest frequency of runs of homozygosity (ROH islands) occurrence across all animals per group; Figure S1. Distribution of inbreeding coefficients (Froh) based on runs of homozygosity (ROH) for each chromosome across groups.
