# Supplementary material for: Selection and Drift: A Comparison between Historic and Recent Dutch Friesian Cattle and Recent Holstein Friesian Using WGS Data
Source: Animals (Basel). 2022 Jan 29;12(3):329. doi: 10.3390/ani12030329 (PMC8833835; doi:10.3390/ani12030329)
Supplement: Supplementary file 1 [file animals-12-00329-s001.zip › Additional file 3-Figure S1.pdf]

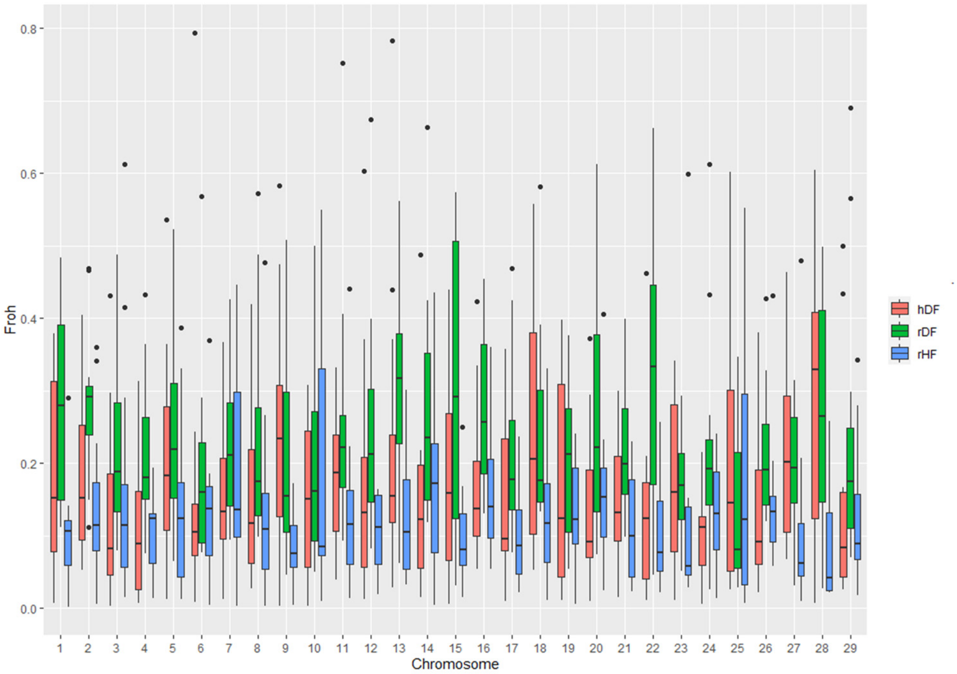

Figure S1. Distribution of inbreeding coefficients (Froh) based on runs of homozygosity (ROH) for each chromosome across groups.
