# Supplementary material for: Selection and Drift: A Comparison between Historic and Recent Dutch Friesian Cattle and Recent Holstein Friesian Using WGS Data
Source: Animals (Basel). 2022 Jan 29;12(3):329. doi: 10.3390/ani12030329 (PMC8833835; doi:10.3390/ani12030329)
Supplement: Supplementary file 1 [file animals-12-00329-s001.zip › Additional file 1-Table S1.pdf]

Table S1. Differentiated genomic regions (ZFst > 8) across autosomal chromosomes for the hDF versus rDF population, the hDF versus rHF population and the rDF versus rHF population

Group	BTA	Start bp	End bp	# SNPs	Weighted Fst	Mean Fst	ZFst
hDF-rDF	1	19,240,001	19,280,000	137	0.31568	0.28208	8.11830
	1	19,260,001	19,300,000	124	0.31381	0.27870	8.01900
	1	53,460,001	53,500,000	87	0.36497	0.29305	8.44001
	1	96,340,001	96,380,000	217	0.44682	0.27948	8.04180
	1	96,500,001	96,540,000	265	0.44624	0.39731	11.49949
	1	96,520,001	96,560,000	227	0.46864	0.42164	12.21353
	1	96,540,001	96,580,000	223	0.47283	0.40570	11.74590
	1	96,560,001	96,600,000	172	0.37235	0.29707	8.55815
	1	98,780,001	98,820,000	118	0.39176	0.33638	9.71162
	1	98,800,001	98,840,000	202	0.49627	0.45689	13.24799
	1	98,820,001	98,860,000	157	0.51724	0.44645	12.94163
	1	101,260,001	101,300,000	194	0.35024	0.28333	8.15487
	2	72,180,001	72,220,000	183	0.39509	0.32438	9.35949
	2	72,540,001	72580,000	93	0.49024	0.40873	11.83473
	2	72,560,001	72,600,000	91	0.54348	0.51100	14.83587
	2	72,580,001	72,620,000	48	0.50611	0.40961	11.86037
	2	72,640,001	72,680,000	22	0.35211	0.28085	8.08215
	2	75,800,001	75,840,000	314	0.43258	0.37650	10.88895
	2	75,820,001	75,860,000	346	0.44958	0.39025	11.29253
	2	129,340,001	129,380,000	253	0.38533	0.31834	9.18231
	2	129,360,001	129,400,000	179	0.37822	0.30326	8.73970
	3	79,120,001	79,160,000	60	0.36410	0.30461	8.77946
	5	51,480,001	51,520,000	254	0.50590	0.32407	9.35045
	5	51,500,001	51,540,000	193	0.50249	0.36656	10.59733
	5	51,520,001	51,560,000	141	0.44374	0.34914	10.08609
	5	92,080,001	92,120,000	170	0.39627	0.29265	8.42836
	7	101,560,001	101,600,000	238	0.34233	0.27871	8.01918
	11	82,960,001	83,000,000	57	0.36766	0.33488	9.66752
	12	74,100,001	74,140,000	351	0.31689	0.29052	8.36591
	13	47,680,001	47,720,000	193	0.36615	0.28172	8.10751
	13	47,700,001	47,740,000	110	0.41587	0.35078	10.13427
	13	47,720,001	47,760,000	76	0.41434	0.35157	10.15734
	13	47,740,001	47,780,000	54	0.40885	0.32184	9.28501
	13	47,760,001	47,800,000	122	0.43150	0.34788	10.04914
	13	47,780,001	47,820,000	218	0.41812	0.28201	8.11607
	17	49,540,001	49,580,000	304	0.36116	0.29127	8.38786
	17	49,560,001	49,600,000	276	0.38671	0.34291	9.90312
	20	33,400,001	33,440,000	254	0.31833	0.28849	8.30643
hDF-rHF	4	44,660,001	44,700,000	280	0.67762	0.58235	8.58204
	4	44,680,001	44,720,000	230	0.67566	0.58614	8.64437
	8	69,680,001	69,720,000	142	0.68894	0.57327	8.43257
	11	9100,,001	9,140,000	274	0.68436	0.57653	8.48624

	16	9,880,001	9,920,000	374	0.62138	0.56617	8.31578
	16	73,540,001	73,580,000	177	0.65779	0.55323	8.10270
	19	55,020,001	55,060,000	195	0.70326	0.58081	8.55658
	20	49,720,001	49,760,000	334	0.74010	0.57479	8.45753
	28	15,160,001	15,200,000	104	0.68975	0.55687	8.16272
rDF-rHF	1	101,260,001	101,300,000	209	0.83254	0.62961	8.29086
	16	9,880,001	9,920,000	374	0.74811	0.68746	9.14869
	20	28,840,001	28,880,000	125	0.74263	0.61200	8.02983
	22	52,800,001	52,840,000	264	0.79243	0.65628	8.68630
	22	52,820,001	52,860,000	308	0.79564	0.69629	9.27954
	22	52,840,001	52,880,000	298	0.76699	0.67055	8.89788
	24	44,160,001	44,200,000	213	0.76937	0.63128	8.31567
