# Supplementary material for: Selection and Drift: A Comparison between Historic and Recent Dutch Friesian Cattle and Recent Holstein Friesian Using WGS Data
Source: Animals (Basel). 2022 Jan 29;12(3):329. doi: 10.3390/ani12030329 (PMC8833835; doi:10.3390/ani12030329)
Supplement: Supplementary file 1 [file animals-12-00329-s001.zip › Additional file 2-Table S2.pdf]

**Table S2**. Genomic regions with the highest frequency of runs of homozygosity (ROH islands) occurrence across all animals per group.

| Group | BTA | # SNPs | Start bp | End bp | Genes |
|---|---|---|---|---|---|
| hDF | 1 | 675 | 106,645,514 | 106,908,592 | ARL14, ENSBTAG00000049656, ENSBTAG00000049943, PPM1L |
| | 7 | 190 | 49,691,470 | 49,900,760 | bta-mir-2459, CDC25C, GFRA3. EGR1, ENSBTAG00000052249, ENSBTAG00000054382, ETF1, FAM53C, HSPA9, KDM3B, REEP2, SLBP2, SNORD63 |
| | 8 | 27 | 61,211,538 | 61,315,643 | ZCCHC7 |
| | 8 | 19 | 61,393,975 | 61,449,116 | ZCCHC7 |
| | 11 | 644 | 79,029,348 | 79,282,225 | |
| | 11 | 798 | 99,199,671 | 99,647,165 | bta-mir-12056, CRAT, DOLK, DOLPP1, ENDOG, ENSBTAG00000036099, ENSBTAG00000055215, IER5L, KYAT1, LRRC8A, MIGA2, NUP188, PHYHD1, PKN3, PTPA, SET, SH3GLB2, SPOUT1, TBC1D13, U6, ZDHHC12, ZER1 |
| | 14 | 410 | 23,116,668 | 23,409,878 | bta-mir-12050, CHCHD7, CLCN6, ENSBTAG00000045097, ENSBTAG00000049644, ENSBTAG00000051516, ENSBTAG00000053852, ENSBTAG00000054153, KIAA2013, LYN, MFN2, MIIP, MOS, MTHFR, NPPA, NPPB, PLAG1, PLOD1, RPS20, U1, U5 |
| | 16 | 937 | 41,624,790 | 41,939,418 | AGTRAP |
| | 16 | 434 | 41,939,940 | 42,292,997 | AGTRAP, DISP3, DRAXIN, FBXO2, FBXO44, FBXO6, MAD2L2 |
| | 16 | 250 | 42,299,812 | 42,353,521 | ENSBTAG00000049854 |
| | 18 | 99 | 14,627,147 | 14,842,392 | DBNDD1, DEF8, ENSBTAG00000038051, ENSBTAG00000055124, FANCA, GAS8, MC1R, SPIRE2, TCF25, TUBB3, U1 |
| | 21 | 350 | 7,412,437 | 7,604,608 | LRRC28, SYNM, TTC23 |
| | 26 | 151 | 506,033 | 691,845 | UBE2D1 |
| rDF | 1 | 1071 | 99,426,412 | 99,801,506 | GOLIM4, LRRC77, PDCD10, SERPINI1 |
| | 1 | 2073 | 100,958,315 | 101,324,016 | |
| | 1 | 757 | 106,577,808 | 106,908,593 | ARL14, ENSBTAG00000049656, ENSBTAG00000049943, ENSBTAG00000055054, PPM1L |
| | 1 | 760 | 112,795,420 | 113,163,480 | |
| | 2 | 1227 | 4,280,885 | 4,580,659 | ENSBTAG00000050981, SAP130, UGGT1 |
| | 2 | 23 | 72,172,782 | 72,177,543 | ENSBTAG00000019464 |
| | 2 | 932 | 72,226,163 | 72,51,6011 | ENSBTAG00000050089 |
| | 2 | 478 | 106,329,099 | 106,642,920 | BCS1L, CATIP, CNOT9, CTDSP1, MIR26B, PLCD4, PNKD, RNF25, SLC11A1, STK36, USP37, VIL1, ZNF142 |
| | 3 | 1363 | 8,713,303 | 9,501,436 | ATP1A4, bta-mir-11983, CASQ1, CD244, CD48, CD84, COPA, DCAF8, ENSBTAG00000010458, ENSBTAG00000015741, ENSBTAG00000051438. ENSBTAG00000052819, LY9, NCSTN, PEA15, PEX19, SLAMF1, SLAMF6, SLAMF7, U6, VANGL2 |

| | | | | |
|---|---|---|---|---|
| 7 | 1116 | 45,793,481 | 46,384,291 | 5S_rRNA, bta-mir-2285di, C7H5orf24, CAMLG, CDKL3, CDKN2AIPNL, DDX46, ENSBTAG00000050332, JADE2, PCBD2, PPP2CA, SAR1B, SEC24A, SKP1 ,TCF7, TXNDC15, U6, UBE2B |
| 7 | 505 | 49,393,699 | 49,908,959 | BRD8, bta-mir-2459, CDC23, CDC25C, EGR1, ENSBTAG00000044792, ENSBTAG00000044864, ENSBTAG00000054382, ENSBTAG00000054560, ETF1, FAM13B, FAM53C, GFRA3, HSPA9, KDM3B, KIF20A, NME5, PKD2L2, REEP2, SLBP2, SNORD63, WNT8A |
| 7 | 1018 | 50,027,040 | 50,857,210 | 5S_rRNA, bta-mir-1949, CTNNA1, DNAJC18, ECSCR, ENSBTAG00000004415, ENSBTAG00000046926, LRRTM2, MATR3, MZB1, PAIP2, PROB1, SIL1, SLC23A1, SMIM33, SNORA74, SPATA24, STING1, UBE2D2 |
| 7 | 456 | 51,413,326 | 51,746,693 | APBB3, CYSTM1, EIF4EBP3, ENSBTAG00000010871, ENSBTAG00000054857, HBEGF, PFDN1, SLC35A4, SLC4A9, SRA1, U6 |
| 8 | 131 | 61,209,017 | 61,451,866 | ZCCHC7 |
| 8 | 62 | 72,722,223 | 72,785,824 | DOCK5, GNRH1, KCTD9 |
| 8 | 1880 | 106,084,876 | 106,413,598 | ASTN2 |
| 10 | 68 | 73,293,126 | 73,321,625 | |
| 10 | 371 | 73,333,656 | 73,689,533 | ENSBTAG00000047000, PRKCH, TMEM30B |
| 11 | 1026 | 17,908,878 | 18,189,921 | |
| 11 | 603 | 99,298,621 | 99,647,165 | bta-mir-12056, CRAT, DOLK, DOLPP1, ENDOG, ENSBTAG00000036099, ENSBTAG00000055215, IER5L, KYAT1, LRRC8A, MIGA2, NUP188, PHYHD1, PTPA, SH3GLB2, SPOUT1 |
| 12 | 152 | 59,667,684 | 59,712,947 | |
| 13 | 857 | 7,475,527 | 7,750,096 | ENSBTAG00000048850, SEL1L2 |
| 13 | 806 | 26,545,252 | 26,904,105 | ENSBTAG00000047440, FZD8, GAD2, GJD4, MYO3A |
| 13 | 35 | 48,965,632 | 48,992,476 | |
| 14 | 675 | 23,021,556 | 23,409,878 | TMEM68, CHCHD7, ENSBTAG00000045097, ENSBTAG00000054153, LYN, PLAG1, RPS20, TGS1, U1 |
| 14 | 1230 | 34,842,119 | 35,402,846 | EYA1 |
| 14 | 513 | 39,799,617 | 40,153,775 | ZFHX4 |
| 15 | 541 | 35,159,302 | 35,422,793 | ENSBTAG00000032859, NUCB2, PIK3C2A, RPS13, SNORD14 |
| 16 | 864 | 25,686,447 | 25,882,406 | |
| 16 | 467 | 41,809,486 | 42,007,254 | AGTRAP, bta-mir-12050, CLCN6, DRAXIN, ENSBTAG00000049644, ENSBTAG00000051516, ENSBTAG00000053852, FBXO6, MAD2L2, MTHFR, NPPA, NPPB |
| 16 | 149 | 42,009,038 | 42,171,425 | DISP3, FBXO2, FBXO44, FBXO6 |
| 16 | 139 | 42,188,151 | 42,292,997 | DISP3 |
| 16 | 331 | 44,367,358 | 44,489,927 | CA6, ENSBTAG00000034323 |
| 17 | 16 | 34,921,218 | 34,927,099 | |

| | | | | | |
|---|---|---|---|---|---|
| | 18 | 543 | 14,271,722 | 14,713,902 | ANKRD11, CDH15, CDK10, CHMP1A, CPNE7, DPEP1, ENSBTAG00000042786, ENSBTAG00000054975, FANCA, MC1R, RPL13, SLC22A31, SPATA2L, SPATA33, SPG7, SPIRE2, TCF25, TUBB3, VPS9D1, ZNF276 |
| | 20 | 1416 | 71,449,101 | 71,891,907 | 5S_rRNA, AHRR, CCDC127, CEP72, ENSBTAG00000026527, ENSBTAG00000052982, EXOC3, LRRC14B, PDCD6, SDHA, SLC9A3, TPPP, U6 |
| | 21 | 125 | 851,400 | 1,098,021 | |
| | 21 | 436 | 2,092,906 | 2,449,109 | bta-mir-11995, ENSBTAG00000048764, ENSBTAG00000052727, ENSBTAG00000053856, SNORD115, SNORD116, UBE3A |
| | 22 | 509 | 49,798,979 | 50,015,556 | C22H3orf18, CACNA2D2, CYB561D2, NPRL2, RASSF1, ZMYND10 |
| | 22 | 469 | 51,519,621 | 51,693,926 | CAMP, CATHL2, CATHL3, CATHL4, CATHL5, CATHL6, CDC25A, ENSBTAG00000016153, ENSBTAG00000031594, ENSBTAG00000039879, ENSBTAG00000052903, NME6 |
| | 24 | 661 | 30,824,756 | 31,056,263 | ENSBTAG00000050912 |
| | 24 | 49 | 3,1057,360 | 31,121,134 | |
| | 26 | 582 | 16,337 | 719,883 | ENSBTAG00000046109, ENSBTAG00000055208, OR5D18, UBE2D1 |
| | 26 | 1029 | 22,459,087 | 23,130,149 | 5S_rRNA, ACTR1A, ARMH3, bta-mir-146b, CUEDC2, ELOVL3, ENSBTAG00000051094, FBXL15, FGF8, GBF1, HPS6, KCNIP2, LDB1, MFSD13A, NFKB2, NOLC1, NPM3, OGA, PITX3, PPRC1, PSD, SNORD22, SUFU |
| | 26 | 697 | 41,573,150 | 41,879,622 | ATE1, ENSBTAG00000048561, ENSBTAG00000049142, NSMCE4A, TACC2 |
| rHF | 8 | 1451 | 105,893,347 | 106,205,184 | ASTN2 |
| | 8 | 654 | 106,207,079 | 106,324,101 | ASTN2 |
| | 14 | 536 | 22,787,828 | 22,887,796 | XKR4 |
| | 14 | 17 | 22,999,212 | 23,004,758 | |
| | 16 | 516 | 44,888,195 | 45,091,226 | bta-mir-2285ck, RERE, SLC45A1 |
| | 19 | 551 | 52,312,182 | 52,611,649 | CARD14, CCDC40, EIF4A3, ENSBTAG00000017233, ENSBTAG00000030186, ENSBTAG00000053071, GAA, Metazoa_SRP, SGSH, SLC26A11, TBC1D16 |
